# Clinical relevance of positively determined myositis antibodies in rheumatology: a retrospective monocentric analysis

**DOI:** 10.1186/s13075-024-03368-9

**Published:** 2024-07-16

**Authors:** Falk Schumacher, Maximilian Zimmermann, Malte Kanbach, Wigbert Schulze, Maximilian Wollsching-Strobel, Doreen Kroppen, Sarah Bettina Stanzel, Daniel Majorski, Wolfram Windisch, Johannes Strunk, Melanie Berger

**Affiliations:** 1 Department of Rheumatology, Krankenhaus Porz am Rhein, Cologne, Germany; 2 Department of Pneumology, Kliniken der Stadt Köln, Cologne, Germany; 3 Faculty of Health/School of Medicine, Witten/Herdecke University, Witten, Germany; 4 Wisplinghoff Medical Laboratories, Cologne, Germany

**Keywords:** Myositis-specific autoantibodies, Myositis-associated autoantibodies, Clinical phenotypes, Inflammatory rheumatic diseases, Idiopathic inflammatory myopathies

## Abstract

**Background:**

The increased availability of myositis autoantibodies represents new possibilities and challenges in clinical practice (Lundberg IE, Tjärnlund A, Bottai M, Werth VP, Pilkington C, de Visser M, et al. 2017 European League Against Rheumatism/American College of Rheumatology classification criteria for adult and juvenile idiopathic inflammatory myopathies and their major subgroups. Ann Rheum Dis. 2017;76:1955–64. 10.1136/annrheumdis-2017-211468.). The aim of this study was to perform a retrospective data analysis of patient cases with positive myositis autoantibodies to analyse their significance in routine rheumatology practice.

**Methods:**

A monocentric analysis of all the orders used to determine myositis autoantibodies from July 2019 to May 2022 in the Department of Rheumatology, Krankenhaus Porz am Rhein, Cologne, Germany, was carried out.

**Results:**

In the defined time interval, a total of 71,597 laboratory values for the antibodies mentioned above were obtained. A total of 238 different positive autoantibodies ​​were detected in 209 patients. Idiopathic inflammatory myopathy was diagnosed in 37 patients (18%), and inflammatory rheumatic diseases other than idiopathic inflammatory myopathy were diagnosed in 90 patients (43%). No inflammatory rheumatic disease was diagnosed in 82 patients (39%). General clusters of clinical manifestations were observed.

**Conclusions:**

In our cohort, we were able to show that a relevant proportion of patients with positive myositis antibodies did not have idiopathic inflammatory myopathies or inflammatory rheumatic diseases. This finding indicates the importance of myositis autoantibodies in this group of patients. However, further studies on the course of symptoms and examination results in patients without inflammatory rheumatic diseases and with positive myositis antibodies are necessary.

**Supplementary Information:**

The online version contains supplementary material available at 10.1186/s13075-024-03368-9.

## Background

Idiopathic inflammatory myopathies (IIMs) are a heterogeneous group of inflammatory rheumatic diseases (IRDs). On the basis of the different clinical manifestations and extended antibody diagnostic results, a differentiated classification of anti-synthetase syndrome (ASS), dermatomyositis (DM), polymyositis (PM), overlap-myositis (OM), immune-mediated necrotizing myopathy (IMNM) and inclusion body myositis (IBM) is possible [1].

Myositis antibodies can be detected in more than 60% of patients with IIMs [2]. With regard to their diagnostic accuracy, myositis antibodies can be divided into myositis-specific antibodies (MSAs) and myositis-associated antibodies (MAAs) [3]. The following antibodies are summarized as MSAs: SRP, Mi-2α, Mi-2β, TIF1-γ, MDA5, NXP2, SAE, EJ, OJ, PL12, PL7, Jo-1, HMGCR, and cN1A. Anti-PM-Scl 75, anti-PM-Scl 100, U1RNP, Ku, and Ro52 are described as MAAs [4, 5]. By definition, MSAs with a specificity of approximately 90% are often involved in key processes in the cell biology of IIMs [6]. MAAs can be detected in 50% of myositis patients and are considered to be less disease-specific and are often associated with overlap myositis [7].

There are large cohorts in the literature, such as EuroMyositis, describing the distribution and clinical association of MSAs/MAAs in cohorts of patients with confirmed IIMs [2, 8]. In another study, all requested MSAs/MAAs from all Dutch patients were analysed. Patients who did not have IIM were considered healthy controls [9]. In addition, other smaller monocentric cohorts in which all myositis antibodies were analysed over a defined period of time have already been described [10, 11].

For everyday rheumatology, however, the significance of positive MSAs/MAAs in patients with other IRDs or previously undiagnosed IRDs is also unclear. Ultimately, we would like to gain knowledge about the relevance of myositis antibodies in patients in whom a clear diagnosis of IIM could not be made in the clinical practice of rheumatology. With this goal in mind, an initially monocentric register was created in this work, which lists all patients for whom a myositis antibody was requested by a rheumatologist. In this first step, the differentiated myositis antibody status, demographic data, diagnoses, clinical phenotypes, and therapeutic courses of the patients in whom the defined MSA/MAA was determined were analysed in more detail.

Due to the inadequate evidence, however, only the Jo-1 antibody could be included in the current EULAR/ACR classification criteria (2017) [12]. The analysis of further current studies showed that the addition of other myositis antibodies should be sought in criteria. The importance of taking a closer look at the clinical phenotype such as skin changes is also highlighted [13]. With regard to the occurrence of Ro52 antibodies, there are data on the clinical relevance in patients with ASS regarding to a higher probability of lung involvement, so that we already have a clinical guideline for the interpretation of this MAA in this area too [14]. Our database was developed to obtain further data on the clinical phenotypes and diagnostic and prognostic relevance of the other MSAs and MAAs in rheumatological clinical practice.

## Methods

### Study design

A retrospective monocentric analysis of all the orders used to determine MSA or MAA incidence from July 2019 to May 2022 in the inpatient and outpatient sectors in the Department of Rheumatology, Krankenhaus Porz am Rhein, Cologne, Germany, was carried out for this study. These data were collected from our monocentric register, which was created in 2022 and lists all patients for whom a myositis antibody was requested by a rheumatologist. In order to be able to investigate clinical courses, a systematic collection of all information documented in the files was carried out. The study was approved by the ethics committee of the University of Witten/Herdecke, Germany (Ref: 153/2022).

### Laboratory tests

All the MSA and MAA results included in this analysis were determined by an external laboratory (Wisplinghoff Medical Laboratories, Cologne, Germany). Sera were tested with a Euroline Autoimmune Inflammatory Myopathies 16 Ag IgG-Immunoblot (Euroimmun, Lübeck, Germany) coated with the following 16 MSA and MAA- antigens: Mi-2α, Mi-2ß, TIF1γ, MDA5, NXP2, SAE1, Ku, PM-Scl100, PM-SCL75, Jo-1, SRP, PL-7, PL-12, EJ, OJ, Ro52 and control. In the first step, the test strip was incubated with a diluted serum sample. If the sample contained specific antibodies, they were bound to the antigens. In the next step, an alkaline phosphatase (AP)-labelled antibody (conjugate), which binds to the specific antibodies, was added. The alkaline phosphatase catalysed a colour reaction with the subsequently added nitro blue tetrazolium chloride/5- bromo-4-chloro-3-indolyl phosphate (NBT/BCIP). If specific antibodies were present in the patient sample, a dark line appeared at the respective antigen position. The intensity of the resulting staining was proportional to the antibody concentration in the sample. The EUROLineScan software was used to evaluate the test results. For this study, the following antigens were included in the evaluation process: EJ, PL-7, OJ, PL-12, Mi-2α, TIF1γ, MDA5, SAE, NXP2, SRP, Ku, PM-Scl100 and PM-Scl75.

Redundant requests, as defined by repeated determinations leading to identical results in each patient, were excluded weakly positive results were also excluded. Other studies have shown that the association between MSAs or MAAs and IIMs are much greater for patients with a clearly positive antibody level than for those with a weakly positive level. [9]

For this study, only the data of patients in whom at least one MSA or MAA was positive were evaluated. Additionally, patients with Jo-1 antibodies were excluded due to the relatively high level of evidence. A total of 13 different myositis antibody subgroups were examined: EJ, PL7, OJ, PL12, Mi-2, TIF1γ, MDA5, SAE, NXP2, SRP, Ku, PM-Scl100, and PM-Scl75.

### Collected patient data

For all patients whose one of the 13 antibodies described above was detected, the patient files were analysed in full by the same person up to May 2022. Demographic data such as sex and age were documented. The first day of the positively determined MSA/MAA was chosen for calculating age.

The main rheumatological diagnoses and relevant secondary diagnoses, such as the occurrence of malignancies, were documented for each patient from the doctor’s letters from the rheumatology clinic. Cancer-associated myositis is defined as the development of both cancer and IIM within a 3-year period [15]. Since our cohort also includes patients without IIM, the occurrence of a malignancy was documented within a 3-year period of the first positive myositis antibody determination. This study involved detailed and systematic analyses of the symptoms that were documented in the clinic. Symptoms that could be evaluated as part of a skin manifestation in the context of an IIM were documented for each patient. IIM-related skin changes included rash, Gottron signs and papules, heliotrope rash, pruritus, alopecia, peripheral ödema, mechanic`s hands, puffy hands, digital ulcera, periungual telangiectasias and sclerodactyly. In addition, the occurrence of Raynaud’s syndrome myalgia/muscle weakness, arthralgia, arthritis, dyspnoea at rest/on exertion, fever > 38 °C, fatigue and weight loss was analysed using the available data. All therapies that the patient had received in his medical history with a rheumatological indication were included.

The following therapies were included in the analysis: methotrexate, chloroquine/hydroxychloroquine, sulfasalazine, azathioprine, rituximab, mycophenolate mofetil, cyclophosphamide, leflunomide, baricitinib, upadacitinib, tofacitinib, filgotinib, adalimumab, certolizumab, golimumab, infliximab, etanercept, secukinumab, ixekizumab, belimumab, abatecept, apremilast, immunoglobulins, anakinra, canakinumab, guselkumab, ustekinumab, tacrolimus, ciclosporin and voclosporin.

### Diagnostic groups

For further evaluation of the data, the patients were divided into three diagnostic groups: IIM, other IRD and no IRD. The first group, IIM, includes anti-synthetase syndrome (ASS), dermatomyositis (DM), polymyositis (PM), overlap-myositis (OM), immune-mediated necrotizing myopathy (IMNM) and inclusion body myositis (IBM). The second group, other IRD, was defined on the basis of the following diagnosis: systemic sclerosis (SSc), systemic lupus erythematosus (SLE), undifferentiated connective tissue disease (UCTD), rheumatoid arthritis (RA), spondyloarthritis (SPA) and other IRDs as giant cell arteritis, cryoglobulinaemic vasculitis, Behçet’s disease, granulomatosis with polyangiitis, microscopic polyangiitis, eosinophilic granulomatosis with polyangiitis, primary sjogren syndrome, mixed connective tissue disease and interstitial pneumonia with autoimmune features. All the other main diagnoses were assigned to the group with no IRD.

### Statistical analysis

Due to the heterogeneous group described here, the descriptive presentation of the data was placed at the forefront of the analysis. The data were described by measures of central tendency (mean) and dispersion (standard deviation (SD)). All the statistical analyses and figures were generated using Microsoft Excel version 2307.

## Results

### Distribution of the examined antibodies

In the defined time interval from July 2017 to May 2022, a total of 71,597 determinations of individual myositis antibodies were determined by the laboratory of our centre. Of these determinations, 1446 tests were positive. After excluding duplicate determinations of the same antibody and after excluding the Jo-1 antibody, 236 values ​​were found to be relevant. The defined MSAs/MAAs where determined in 209 patients. The distribution of antibodies in the cohort is shown in Table 1. Patients with more than one positive antibody were not excluded. The number of positively determined MAAs was greatest (Pm-Scl75: *n* = 54, PMScl75: *n* = 44 and Ku: *n* = 41). Among the MSA patients, Pl7 antibodies (*n* = 24) were the most common, followed by Mi-2, TIF1γ, Pl12, NXP2 and SRP. SAE, OJ, MDA5 and EJ represented rather smaller subgroups, occurring in 4 to 7 patients in the cohort.

**Table 1:** Number of patients in whom at least one MSA/MAA was positive (patients with several positive antibodies not excluded). MSA: myositis-specific antibodies, MAA: myositis-associated antibodies

Positive determined antibody	Number of patients in the antibody-groups, *n* (%)
**MSA**	
EJ	4 (1.7)
PL7	24 (10.2)
OJ	7 (3.0)
PL12	12 (5.1)
Mi-2	13 (5.5)
TIF1γ	13 (5.5)
MDA5	5 (2.1)
SAE	7 (3.0)
NXP2	11 (4.7)
SRP	10 (4.2)
**MAA**	
Ku	41 (17.4)
PM-Scl100	44 (18.6)
PM-Scl75	45 (19.1)

Duplicate determinations of different autoantibodies in one patient were performed for 21 patients (10.0%). Sixteen patients (7.7%) had an MSA combined with one or more MAA. The occurrence of 2 or more MSAs is shown in 5 patients (2.4%). In general (MSAs and MAAs) the most frequent was the simultaneous occurrence of 2 antibodies (*n* = 17), followed by 3 different antibodies (*n* = 3). In one patient, 5 different MSAs/MAAs were positively detected (Table 2).

**Table 2:** Frequency of simultaneously positive MSAs/MAAs in the cohort and the diagnoses of the patients. All diagnoses without patient numbers represent *n* = 1 (IIM: idiopathic inflammatory myopathy, other IRD: inflammatory rheumatic disease other than IIM; no IRD: no inflammatory rheumatic disease; ASS: anti-synthetase syndrome; DM: dermatomyositis; PM: polymyositis; OM: overlap-myositis; SSc: systemic sclerosis; SLE: systemic lupus erythematosus; UCTD: undifferentiated connective tissue disease; RA: rheumatoid arthritis; SPA: spondyloarthritis; other: giant cell arteritis, cryoglobulinemic vasculitis, behçet’s disease, microscopic polyangiitis, primary sjögren syndrome, mixed connective tissue disease)

*N* (= 21)	MSA/MAA	Diagnosis
6	PM/Scl-75 + PM/Scl-100	OM (*n* = 4), DM, UCTD
1	PM/Scl-75 + EJ	No IRD
1	PM/Scl-100 + NXP2	DM
1	PM/Scl-100 + Ku	UCTD
1	Ku + OJ	No IRD
2	Ku + PL12	No IRD, OM
2	Ku + PL7	UCTD, no IRD
1	PL7 + PL12	No IRD
1	PL7 + NXP2	No IRD
1	TIF1γ + SAE	OM
1	PM/Scl-75 + PM/Scl-100 + Ku	No IRD
1	PM/Scl-75 + PM/Scl-100 + PL12	OM
1	Mi-2 + TIF1γ + MDA5	RA
1	Ku + OJ + SAE + NXP2 + SRP	UCTD

If all patients in whom more than one of the investigated antibodies was positive were divided into the three diagnostic groups, this group had a relatively greater proportion of IIM patients (IIM, *n* = 9 [42,9%]; other IRD, *n* = 5 [23,8%]; no IRD, *n* = 7 [33,3%]; in contrast to the entire cohort.

### Patient demographics and characteristics

**Fig. 1:**
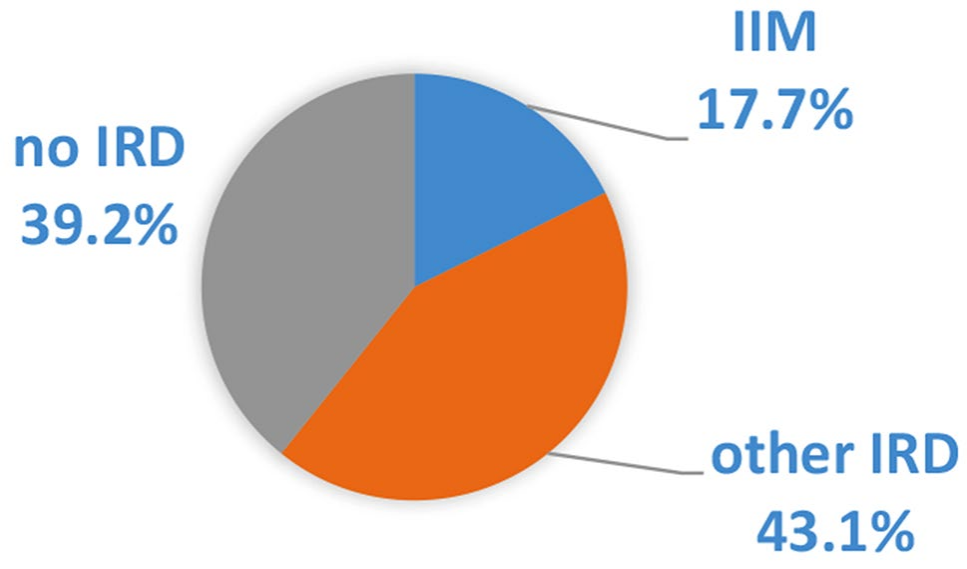
Relative frequency of the diagnostic groups in the whole examined cohort (IIM: idiopathic inflammatory myopathy; other IRD: other inflammatory rheumatic disease than IIM; no IRD: no inflammatory rheumatic disease)

69% of the 209 examined patients were female. The mean (SD) age was 60.5 (+-5.6) years. The patients were divided into three diagnostic groups. 38 patients were diagnosed with idiopathic inflammatory myopathy (18%), 90 patients were diagnosed with IRD other than IIM (43%) and 82 patients (39%) were not diagnosed with an IRD (Fig. 1). In our cohort from everyday practice in a rheumatology clinic, the IIM group represented the smallest group. Patients with other IRDs or without IRDs more frequently existed with a similar number of patients.

Further characterization of the group of IIM and the group of other IRD is shown in Table 3. In the group of IIM the diagnosis of OM was most common, followed by DM and PM, with ASS being diagnosed the least frequently. In the group of other IRD, most patients were diagnosed with rheumatoid arthritis (RA) (*n* = 32) or undifferentiated connective tissue disease (UCTD) (*n* = 25).

**Table 3:** Number of patients in the diagnostic groups (IIM: idiopathic inflammatory myopathy; other IRD: inflammatory rheumatic disease other than IIM; no IRD: no inflammatory rheumatic disease; ASS: anti-synthetase syndrome; DM: dermatomyositis; PM: polymyositis; OM: overlap-myositis; SSc: systemic sclerosis; SLE: systemic lupus erythematosus; UCTD: undifferentiated connective tissue disease; RA: rheumatoid arthritis; SPA: spondyloarthritis; other: giant cell arteritis; cryoglobulinemic vasculitis, behçet’s disease; microscopic polyangiitis; primary sjögren syndrome; mixed connective tissue disease)

Number of patients in the diagnostic groups, *n* = 209 (%)										
IMM, 37 (17.7)				Other IRD, 90 (43.1)						No IRD, 82 (39.2)
DM, 9 (4.3)	PM, 7 (3.3)	ASS, 2 (1.0)	OM, 19 (9.1)	SSc, 5 (2.4)	SLE, 9 (4.3)	UCTD, 25 (12.0)	RA, 32 (15.3)	SPA, 9 (4.3)	Other, 10 (4.8)	No IRD, 82 (39.2)

### MSAs and MAAs in the diagnostic groups

**Fig. 2:**
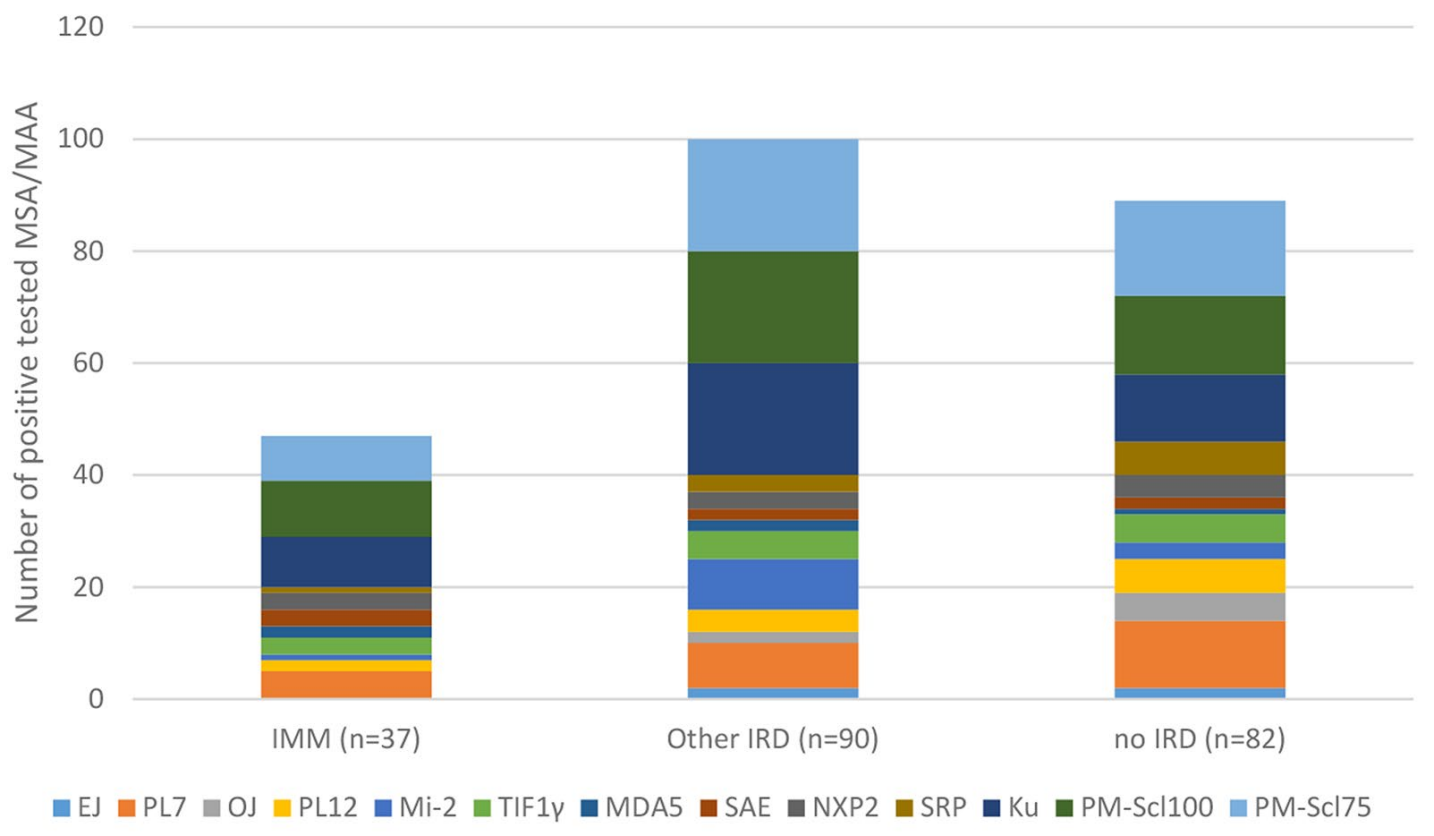
Distribution of MSAs/MAAs and their absolute level of detection in the diagnostic groups (IIM: idiopathic inflammatory myopathy, other IRD: inflammatory rheumatic disease other than IIM; no IRD: no inflammatory rheumatic disease)

According to the absolute numbers of MSAs/MAAs present in the three diagnostic groups, some antibodies did not occur at all in the IIM group, which may also be due to the smaller number of patients in this group (Fig. 2).

According to the relative consideration of the diagnoses in the individual antibody groups, PL7 and PL12 antibodies occurred more frequently in the group with no IRD than in the group with other IRDs, but Mi-2 and MAA (Pm-Scl100, Pm-Scl75 and Ku) occurred more frequently in the group with other IRDs than in the group with no IRD.

**Fig. 3:**
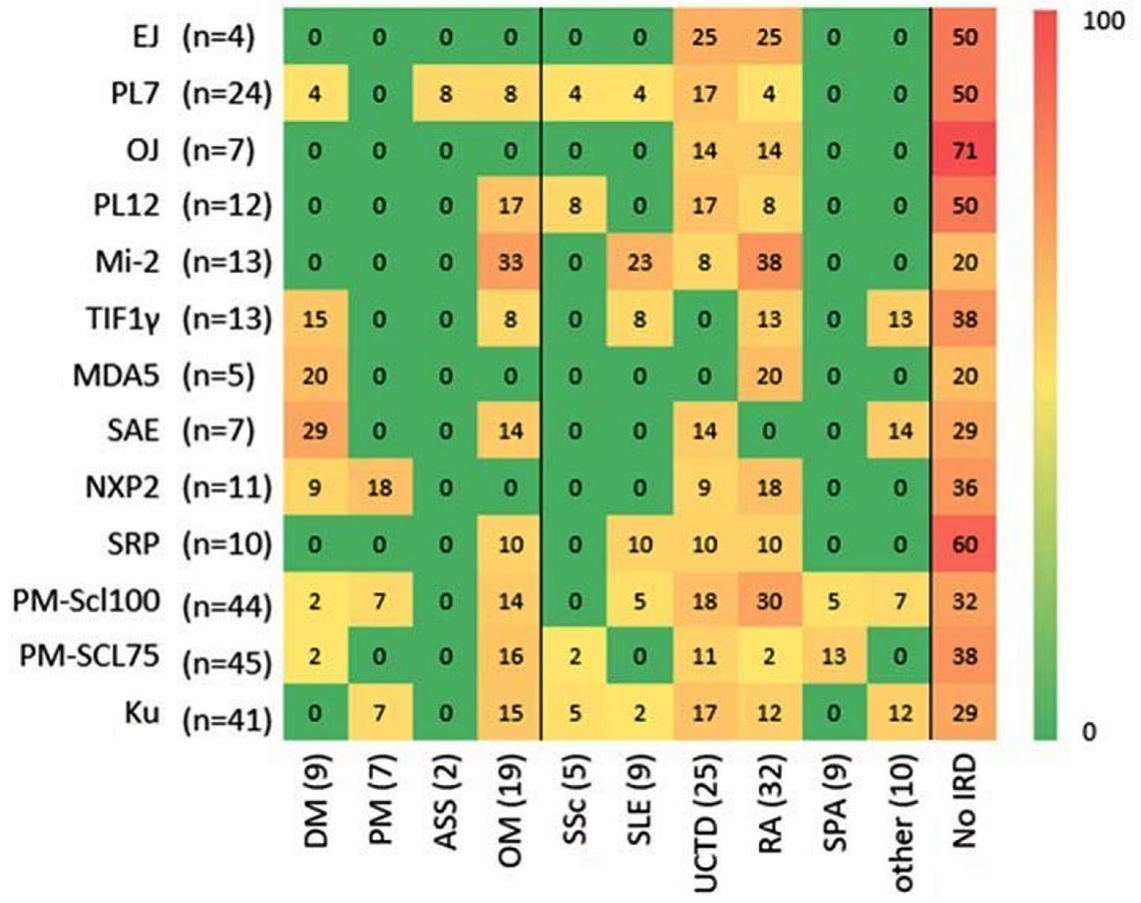
Diagnosis in the antibody subgroups. The distribution of the different diagnoses was visualized in the different antibody subgroups in a heatmap. The numbers in boxes indicate the percentage of patients positive for a particular diagnosis in an antibody subgroup. The numbers of patients within each subgroup are indicated on the right of each antibody and above the diagnosis groups (IIM: idiopathic inflammatory myopathy; other IRD: inflammatory rheumatic disease other than IIM; IRD: no inflammatory rheumatic disease; ASS: anti-synthetase syndrome; DM: dermatomyositis; PM: polymyositis; OM: overlap-myositis; SSc: systemic sclerosis; SLE: systemic lupus erythematosus; UCTD: undifferentiated connective tissue disease; RA: rheumatoid arthritis; SPA: spondyloarthritis; Other: giant cell arteritis, cryoglobulinemic vasculitis; Behçet’s disease, microscopic polyangiitis, primary Sjogren syndrome, mixed connective tissue disease)

Considering the diagnosis groups, it also becomes clear that, in the present setting, there was a relevant proportion of patients in the group with no IRD for each antibody examined. A diagnosis based on the antibody alone is of course not possible, but the diagnostic relevance of MSAs/MAAs in the no IRD group will be shown by the further clinical course in this cohort (Fig. 3). In addition, OJ antibodies and SRP were present in a particularly high proportion of the no IRD group, whereas Mi-2 and SAE are mainly present in the group of other IRD. In the diagnostic subgroups of UCTD and RA, there is also an accumulation of several antibodies. In total, positive RF and/or ACPA were found in 10 of the 32 patients (31%) with a diagnosis of rheumatoid arthritis and positive myositis antibodies. Thus, the majority of patients with RA in our cohort did not have positive RF or ACPA.

A closer look at the IIM group confirmed various tendencies described in the literature. TIF1γ, MDA5 and SAE were increasingly detected in the DM group. The examined MAAs (Pm-Scl100, Pm-Scl75 and Ku) in the IIM group were mainly found in the diagnostic subgroup of OM.

Positive antisynthetase antibodies were determined in 47 patients (23%). According to the doctor’s letter, only 2 patients (1%) were diagnosed with ASS. If the Connors criteria for ASS were applied to our cohort, the diagnosis of ASS could be made in 25 patients (12%) [16].

### Clinical features of the cohort

**Fig. 4:**
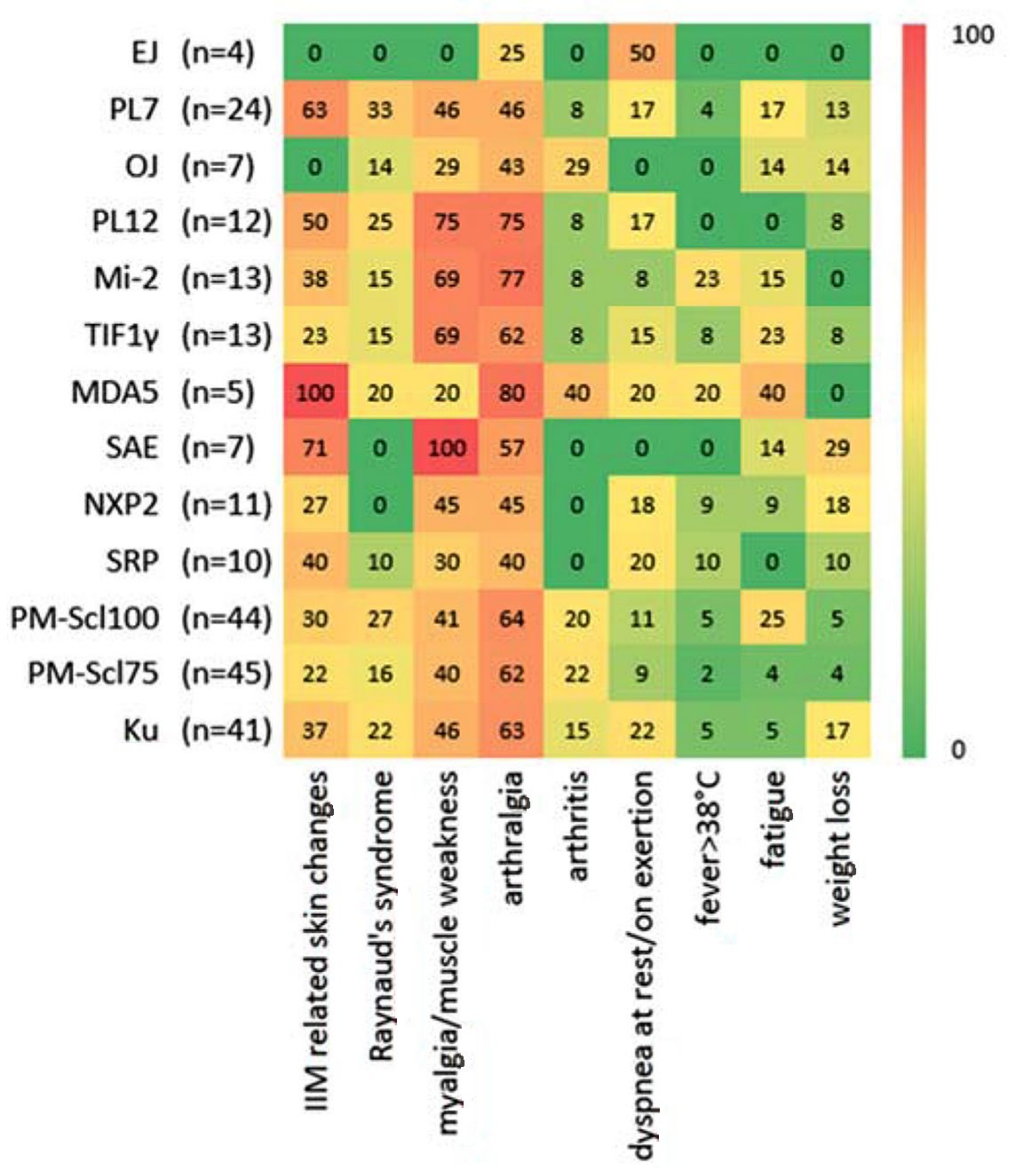
Clinical features of the patients in the antibody subgroups. The distribution of the different clinical symptoms was visualized in the different antibody subgroups in a heatmap. The numbers in boxes indicate the percentage of patients positive for a particular clinical future in the antibody subgroup. The numbers of patients within each subgroup are indicated on the right of each antibody. IIM related skin changes, including rash, Gottron signs and papules, heliotrope rash, pruritus, alopecia, peripheral edema, mechanic hands, puffy hands, digital ulcers, periungual telangiectasias, sclerodactyly were added together

Regarding the clinical features, the present results in our cohort show that there are groups of symptoms that occur in 100% of the patients in the respective antibody groups. This relates to the symptoms of IIM related skin changes in the MDA5-group as well as to the myalgia/muscle weakness in the SAE-group (Fig. 4). However, these groups also included patients from the diagnosis groups no IRD and other IRD. Other symptoms such as arthralgias were present in all diagnostic groups. Arthritis was more common in the OJ, MDA5 and MAA groups. Fever was more common in the dermatomyositis-associated antibody groups MDA5 and Mi2.

### Frequency of malignancy occurrence depending on antibody status

In the present cohort, we observed the greatest relative frequency of malignancies in the SAE (0,43), SRP (0,40) and TIF1γ (0,31) antibody subgroups. Among the MAA and anti-synthetase antibody groups, there was a tendency to be a lower probability of malignancy than among the antibody group, which can be related to dermatomyositis (Table 4).

**Table 4:** Absolute and relative frequency of malignancy occurrence in the antibody subgroups

Antibody subgroup		absolute frequency of malignancies	relative frequency for malignancy
EJ	(*n* = 4)	1	**0.25**
PL7	(*n* = 24)	3	**0.13**
OJ	(*n* = 7)	2	**0.29**
PL12	(*n* = 12)	1	**0.08**
Mi-2	(*n* = 13)	2	**0.15**
TIF1γ	(*n* = 13)	4	**0.31**
MDA5	(*n* = 5)	0	**0**
SAE	(*n* = 7)	3	**0.43**
NXP2	(*n* = 11)	2	**0.18**
SRP	(*n* = 10)	4	**0.40**
PM-Scl100	(*n* = 44)	5	**0.11**
PM-Scl75	(*n* = 45)	4	**0.09**
Ku	(*n* = 41)	6	**0.15**

### Therapy used in the cohort and depending on antibody status

A total of 17 different therapeutic agents were used in our cohort (Supplementary figure S1). A closer look at the immunomodulating therapies used showed that the patient groups treated with methotrexate (*n* = 45) and an antimalarial agent (chlororquine/hydroxychloroquine, *n* = 30) clearly represented the largest groups. Relatively speaking, these two groups included a greater proportion of patients with MAA than with MSA. With other therapies, such as cyclophosphamide, the distribution of the antibodies found in the therapeutic groups was more homogeneous.

## Discussion

The clinical relevance of MSAs/MAAs has been examined in many cohorts consisting of patients who have IMM [2, 8]. Patients who were treated at university hospitals due to serious organ involvement were often included. Health insurance data from large registries are limited by the insufficient specificity of International Classification of Diseases (ICD) codes for patients with IIMs, as the classification system is currently changing [17]. In our registry, patients are classified based on the medical diagnosis in the doctor’s letters and not based on the ICD codes, which represents a closer connection to clinical reality.

According to our cohort, the IIM subgroup had the smallest percentage (17.7%), and the percentages of patients in the no IRD and other IRD groups were similarly large (39.2% and 43.1%, respectively). Therefore, myositis antibodies occur in rheumatological practice to a relevant extent in patients without IIMs. Considering the clinical phenotype, there are also clinical manifestations related to an antibody, which can be observed in all three groups. This finding provides insight into the relevance of these antibodies and the need to reclassify our picture of the importance of these antibodies in these patients.

The data regarding positive MSAs/MAAs and the association with malignant diseases in cohorts, including patients without IIMs, are very limited. When comparing our data with those from IIM cohorts, similar trends regarding the increased occurrence of malignancies in patients with TIF1γ and SAE and the rather low association with malignancy with antisynthetase antibodies were reproduced [18–20].

In our cohort, the occurrence of multiple MSAs/MAAs in one patient was found in 21 cases (10.0%). Other studies have shown multiple occurrence in (19.3%) of IIM patients and (12.5%) of non IRD patients. In addition, as previously described, we were able to confirm the increased cooccurrence of anti-PM/Scl-75 and anti-PM/Scl-100. [9] The occurrence of multiple antibodies in one patient represents a higher probability of the presence of IIMs in our cohort.

Myositis line blot assays show high specificity but low sensitivity. As stated in other studies, we can confirm that MSAs/MAAs play an important role in further elucidation and differentiation of suspected IIMs [21]. This method should not be used for screening for suspected inflammatory myopathy. In our study, the importance of MSAs/MAAs in patients with another type of IRD also became clearer since a specific pattern of symptoms can be recognized even in patients without IIMs. In addition, further follow-up data are needed to clarify the relevance of myositis antibodies in patients who do not have IRD. The group of patients in our cohort was preselected by referral to the rheumatologist and by the decision of the rheumatologist to determine the myositis antibodies. In this group of patients without IRDs, further investigation of the clinical course is certainly useful.

In our everyday clinical practice, all patients with positive myositis antibodies and respiratory symptoms are presented to the pulmonologist, even if they have no other symptoms of CTD. A closer look at the course of this patient without IRD and positive myositis antibodies can provide valuable insights into possible predictive factors for the occurrence of IIM with or without ILD in the future. This could also provide a more precise definition of which patients without IIM the myositis antibodies are relevant for. It is striking that there was still a large quantitative mismatch between the number of determined myositis antibodies and the number of positive results. Better evidence regarding the diagnostic and prognostic significance of MSAs/MAAs could also be helpful in terms of cost-effectiveness. It might then be possible to determine the specific MAAs/MSAs in special clinical constellations.

When comparing the clinical phenotypes of dermatomyositis-associated antibodies in our cohort with those in IIM cohorts, there were both consistent and novel clinical patterns. In our cohort, the previously described increased incidence of arthralgia in patients with MDA5 antibodies was confirmed [22, 23]. The rather amyopathic course of the patients with MDA5 antibodies could also be reproduced in our data. In contrast to what has been described in the literature, patients with TIF1γ or SAE also exhibited more frequent occurrence of myalgia and muscle weakness in our cohort. [24]. The increased occurrence of IIM-related skin changes in patients with SAE antibodies was confirmed in our cohort, whereas this tendency was not as clear in our TIF1γ group [25]. The pattern of Mi-2-positive patients also fits the literature, with frequent occurrence of myalgia and muscle weakness and rare occurrence of dyspnoea [12].

With regard to antisynthetase antibodies, there was a surprisingly low proportion of patients with symptoms of dyspnoea in our cohort, which is certainly also related to the large proportion of patients with no IRD in these groups [26].

From the IMNM group, we examined the SRP antibodies in our group. We were able to reproduce the increased occurrence of muscular symptoms as previously described, although arthralgia and IIM-related skin changes were relatively frequent in our whole cohort [27].

In our group of MAA, as described in the literature, an increased occurrence of Raynaud’s syndrome can be observed, which must be considered in connection with the association with systemic sclerosis [7]. In addition, we also observed frequent IIM-related skin changes and muscular symptoms in this group. The increased occurrence of arthralgias in almost all antibody groups could be due to the selection of patients with one of the most common symptoms for referral to rheumatology.

There is no antibody-specific therapy recommendation for patients with IRD and positive MSAs/MAAs. However, in our cohort, the proportion of patients with MAAs in the groups treated with methotrexate or hydroxychloquine was greater than the proportion of patients with MSA. Other treatments used did not show any clear trends regarding antibody status. The patients were controlled according to organ involvement. From the example of our group treated with cyclophosphamide, it becomes clear that this therapy decision was not made dependent on the antibody status.

Our study has also limitations. First, the study was a single-center study. The retrospective data collection also represents a limitation. However, this creates a base of patient cases that offers the possibility for specific prospective studies. The diagnoses of IIM and other IRDs were taken from the documentation of the treating rheumatologist. A possible correction regarding the classification criteria was not made, which, on the one hand, makes comparability more difficult but, on the other hand, can improve the clinical reference. Using the example of antisynthetase antibodies, a discrepancy in the number of clinical diagnoses of ASS compared to the number of patients who meet the Connors criteria was also found. Due to the retrospective data analysis, we decided against an evaluation based on classification criteria.

Due to the better sensitivity and specificity of clearly positive MSAs/MAAs than weakly positive MSAs/MAAs described in other studies [9], only patients with clearly positive antibodies were included in our study. Every day, clinical practice shows that even weakly positive MSAs/MAAs can have clinical relevance for individual cases, which is also controversial in the literature [2]. During the investigation period of 3 years, an antibody was considered positive if it was positive once. Since the patients were in different phases of treatment, no distinction was made as to whether the antibody was confirmed again in an examination or not. This could therefore also include false positive cases. The exclusion of common myositis antibodies results in poorer comparability. However, this enabled us to carry out a particularly detailed evaluation of the rarer antibodies to increase the available data in this area. Basically in clinical practice a re-testing should be carried out in the case of multiple positive MAA or MSA.

Important strengths of these studies are the direct reference to everyday clinical practice in rheumatology, the relatively high number of antibodies that are otherwise rare, and the detailed evaluation of the clinical features of the respective patients.

## Conclusions

MSA and MAA are playing an increasingly important role in the diagnostics and therapeutic management of IIM. In our cohort, we were able to show that a relevant proportion of patients with positive myositis antibodies did not have idiopathic inflammatory myopathies or inflammatory rheumatic diseases. When looking at all patient groups, overarching clusters of clinical manifestations can be seen. This gives us an indication of the importance of MSA/MAA in patients without IIM. This finding indicates the importance of myositis autoantibodies in this group of patients. However, further studies on the course of symptoms and examination results in patients without inflammatory rheumatic diseases and with positive myositis antibodies are necessary.

### Electronic Supplementary Material

Below is the link to the electronic supplementary material.

Supplementary Material 1

## Data Availability

All the primary data and evaluations on which this study is based are accessible to the investigators at any time in our centre and have been archived for at least 10 years.

## Abbreviations

ASS: Anti-synthetase syndrome
BCIP 5: Bromo-4-chloro-3-indolyl phosphate
DM: Dermatomyositis
IBM: Inclusion body myositis
ICD: International Classification of Diseases
IIMs: Idiopathic inflammatory myopathies
IMNM: Immune-mediates necrotizing myopathie
IRDs: Inflammatory rheumatic diseases
MAAs: Myositis-associates antibodies
MSAs: Myositis-specific antibodies
NBT: Nitro blue tetrazolium chloride
OM: Overlap-myositis
PM: Polymyositis
RA: Rheumatoid arthritis
SD: Standard deviation
SLE: Systemic lupus erythematosus
SPA: Spondyloarthritis
SSc: Systemic sclerosis
UCTD: Infifferentiated connective tissue disease

## References

[1] Tomaras S, Feist E (2023). Myositissyndrome. [Myositis]. Inn Med (Heidelb).

[2] Betteridge Z, Tansley S, Shaddick G, Chinoy H, Cooper RG, New RP (2019). Frequency, mutual exclusivity and clinical associations of myositis autoantibodies in a combined European cohort of idiopathic inflammatory myopathy patients. J Autoimmun.

[3] Ghirardello A, Borella E, Beggio M, Franceschini F, Fredi M, Doria A (2014). Myositis autoantibodies and clinical phenotypes. Auto Immun Highlights.

[4] Mahler M, Miller FW, Fritzler MJ (2014). Idiopathic inflammatory myopathies and the anti-synthetase syndrome: a comprehensive review. Autoimmun Rev.

[5] Palterer B, Vitiello G, Carraresi A, Giudizi MG, Cammelli D, Parronchi P (2018). Bench to bedside review of myositis autoantibodies. Clin Mol Allergy.

[6] Ghirardello A, Bassi N, Palma L, Borella E, Domeneghetti M, Punzi L, Doria A (2013). Autoantibodies in polymyositis and dermatomyositis. Curr Rheumatol Rep.

[7] Iaccarino L, Gatto M, Bettio S, Caso F, Rampudda M, Zen M (2013). Overlap connective tissue disease syndromes. Autoimmun Rev.

[8] Wen L, Chen X, Cheng Q, Nie L, Xu J, Yan T (2022). Myositis-specific autoantibodies and their clinical associations in idiopathic inflammatory myopathies: results from a cohort from China. Clin Rheumatol.

[9] Platteel ACM, Wevers BA, Lim J, Bakker JA, Bontkes HJ, Curvers J (2019). Frequencies and clinical associations of myositis-related antibodies in the Netherlands: a one-year survey of all Dutch patients. J Transl Autoimmun.

[10] Lecouffe-Desprets M, Hémont C, Néel A, Toquet C, Masseau A, Hamidou M (2018). Clinical contribution of myositis-related antibodies detected by immunoblot to idiopathic inflammatory myositis: a one-year retrospective study. Autoimmunity.

[11] Loarce-Martos J, Calvo Sanz L, Garrote-Corral S, Ballester González R, Pariente Rodríguez R, Rita CG (2023). Myositis autoantibodies detected by line blot immunoassay: clinical associations and correlation with antibody signal intensity. Rheumatol Int.

[12] Lundberg IE, Tjärnlund A, Bottai M, Werth VP, Pilkington C, de Visser M (2017). 2017 European League Against Rheumatism/American College of Rheumatology classification criteria for adult and juvenile idiopathic inflammatory myopathies and their major subgroups. Ann Rheum Dis.

[13] Saygin D, Glaubitz S, Zeng R, Bottai M, de Visser M, Dimachkie MM (2024). Performance of the 2017 EULAR/ACR classification Criteria for adult and juvenile idiopathic inflammatory myopathies and their major subgroups: a scoping review. Clin Exp Rheumatol.

[14] Bozzalla-Cassione E, Zanframundo G, Biglia A, Bellis E, Bozzini S, Codullo V (2022). Anti-Ro52 antibodies positivity in antisynthetase syndrome: a single centre cohort study. Clin Exp Rheumatol.

[15] Troyanov Y, Targoff IN, Tremblay J-L, Goulet J-R, Raymond Y, Senécal J-L (2005). Novel classification of idiopathic inflammatory myopathies based on overlap syndrome features and autoantibodies: analysis of 100 French Canadian patients. Med (Baltim).

[16] Connors GR, Christopher-Stine L, Oddis CV, Danoff SK (2010). Interstitial lung disease associated with the idiopathic inflammatory myopathies: what progress has been made in the past 35 years?. Chest.

[17] Lundberg IE, Fujimoto M, Vencovsky J, Aggarwal R, Holmqvist M, Christopher-Stine L (2021). Idiopathic inflammatory myopathies. Nat Rev Dis Primers.

[18] Stockton D, Doherty VR, Brewster DH (2001). Risk of cancer in patients with dermatomyositis or polymyositis, and follow-up implications: a Scottish population-based cohort study. Br J Cancer.

[19] Trallero-Araguás E, Rodrigo-Pendás JÁ, Selva-O’Callaghan A, Martínez-Gómez X, Bosch X, Labrador-Horrillo M (2012). Usefulness of anti-p155 autoantibody for diagnosing cancer-associated dermatomyositis: a systematic review and meta-analysis. Arthritis Rheum.

[20] Ge Y, Lu X, Shu X, Peng Q, Wang G (2017). Clinical characteristics of anti-SAE antibodies in Chinese patients with dermatomyositis in comparison with different patient cohorts. Sci Rep.

[21] Montagnese F, Babačić H, Eichhorn P, Schoser B (2019). Evaluating the diagnostic utility of new line immunoassays for myositis antibodies in clinical practice: a retrospective study. J Neurol.

[22] Hall JC, Casciola-Rosen L, Samedy L-A, Werner J, Owoyemi K, Danoff SK, Christopher-Stine L (2013). Anti-melanoma differentiation-associated protein 5-associated dermatomyositis: expanding the clinical spectrum. Arthritis Care Res (Hoboken).

[23] Best M, Jachiet M, Molinari N, Manna F, Girard C, Pallure V (2018). Distinctive cutaneous and systemic features associated with specific antimyositis antibodies in adults with dermatomyositis: a prospective multicentric study of 117 patients. J Eur Acad Dermatol Venereol.

[24] Hamaguchi Y, Kuwana M, Hoshino K, Hasegawa M, Kaji K, Matsushita T (2011). Clinical correlations with dermatomyositis-specific autoantibodies in adult Japanese patients with dermatomyositis: a multicenter cross-sectional study. Arch Dermatol.

[25] Fiorentino DF, Kuo K, Chung L, Zaba L, Li S, Casciola-Rosen L (2015). Distinctive cutaneous and systemic features associated with antitranscriptional intermediary factor-1γ antibodies in adults with dermatomyositis. J Am Acad Dermatol.

[26] Hervier B, Devilliers H, Stanciu R, Meyer A, Uzunhan Y, Masseau A (2012). Hierarchical cluster and survival analyses of antisynthetase syndrome: phenotype and outcome are correlated with anti-tRNA synthetase antibody specificity. Autoimmun Rev.

[27] Allenbach Y, Benveniste O, Stenzel W, Boyer O (2020). Immune-mediated necrotizing myopathy: clinical features and pathogenesis. Nat Rev Rheumatol.

