# Supplementary material for: Clinical relevance of positively determined myositis antibodies in rheumatology: a retrospective monocentric analysis
Source: Arthritis Res Ther. 2024 Jul 16;26:132. doi: 10.1186/s13075-024-03368-9 (PMC11251291; doi:10.1186/s13075-024-03368-9)
Supplement: Supplementary file 1 — Supplementary Material 1 [file 13075_2024_3368_MOESM1_ESM.docx]

**Supplemental material:**

*Supplementary figure S1: Therapy used in the cohort and positive MSAs and MAAs in the medication subgroups. Immediately following the drug name is the number of patients in our cohort who received that drug. The right panel shows the number of patients with positive MSAs/MAAs in the respective medication groups.*
